# Drummer's Thumb: Ulnar Digital Artery Thrombosis and Aneurismal Change From Repetitive Drumstick Trauma After Carpal-Metacarpal Arthroplasty

**Published:** 2019-06-18

**Authors:** Michael R. Ruebhausen, Nada N. Berry

**Affiliations:** Institute for Plastic Surgery, Southern Illinois University School of Medicine, Springfield

**Keywords:** arterial thrombosis, hypothenar hammer, CMC arthroplasty, drummer, repetitive trauma

## CASE DESCRIPTION

**Figure 1:**
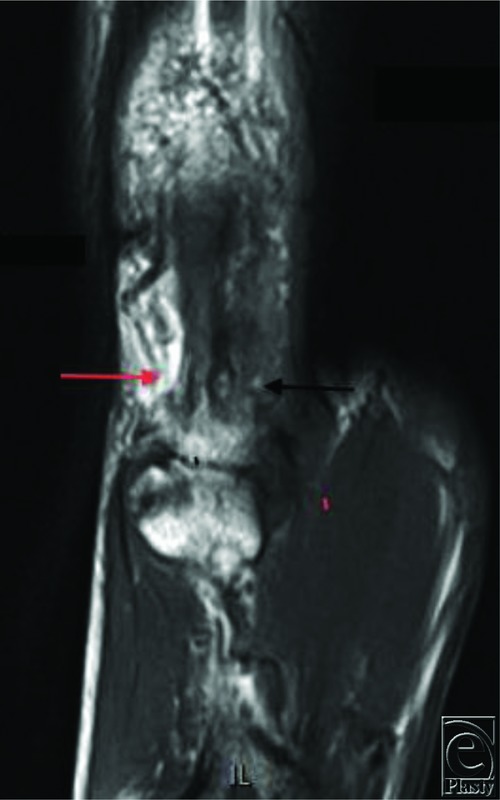
Magnetic resonance imaging of the left thumb. STIR images showing hypointensity of the ulnar digital vessel (black arrow). Note the hyperintense signal of the patent radial digital artery (red arrow). This finding was not considered diagnostic of arterial occlusion.

**Figure 2:**
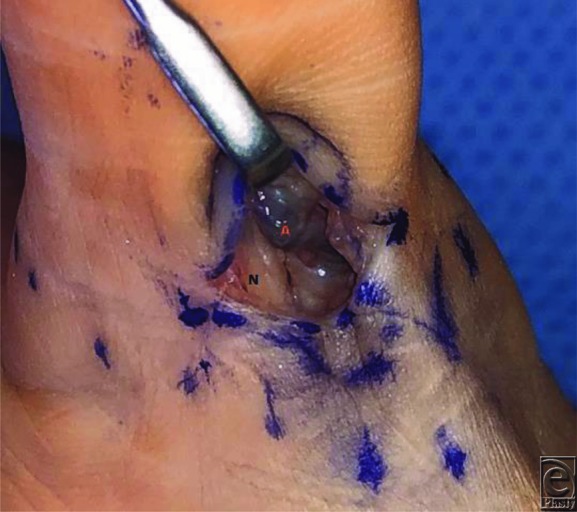
In situ thrombosed ulnar digital artery of the left thumb. Notice the purple discoloration of thrombus as well as the proximal white discoloration and the “corkscrew” conformation of the vessel (A, red). Thrombus was noted in the vessel when it was cut proximally, but there was no backflow even after thrombus removal. The nerve is linear and appears normal (N, black).

**Figure 3:**
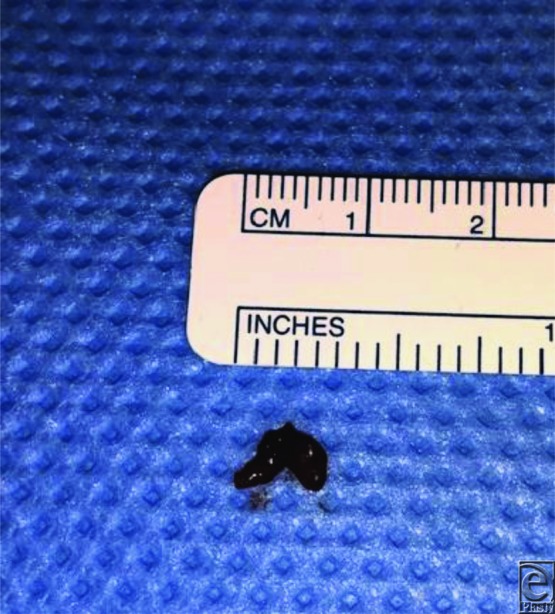
Transected arterial specimen. Note that the corkscrew conformation persisted after transection, suggesting significant intimal damage.

A 60-year-old man presented for evaluation of new-onset thumb pain 4 months after trapeziectomy and carpal-metacarpal (CMC) arthroplasty with suspensionplasty. His primary complaint was a palpable, painful nodule at the ulnar base of the thumb. He experienced occasional shooting pain to the tip of the thumb while playing drums, as he was a semiprofessional drummer. Magnetic resonance imaging (MRI) was ordered, which showed only local soft tissue reaction, likely consistent with scar tissue or local thickening (Fig 1). Surgical exploration revealed a thrombosed corkscrew ulnar digital artery (Fig 2) similar to vessel appearance in hypothenar hammer syndrome (HHS). A digital Allen's test was performed,^1^ which demonstrated vascular patency to the thumb. Excision without reconstruction was elected at this time. The segment that was resected was sent for permanent pathology in formalin (Fig 3). The patient reported symptomatic relief, including while playing drums.

## QUESTIONS

1. What extremity-related diseases are drummers known for?
2. What is the differential for a mass at the base of the thumb?
3. How does trauma-related vasospastic disease usually present?
4. What does this surgical finding represent and who does it affect?

**Figure 4:**
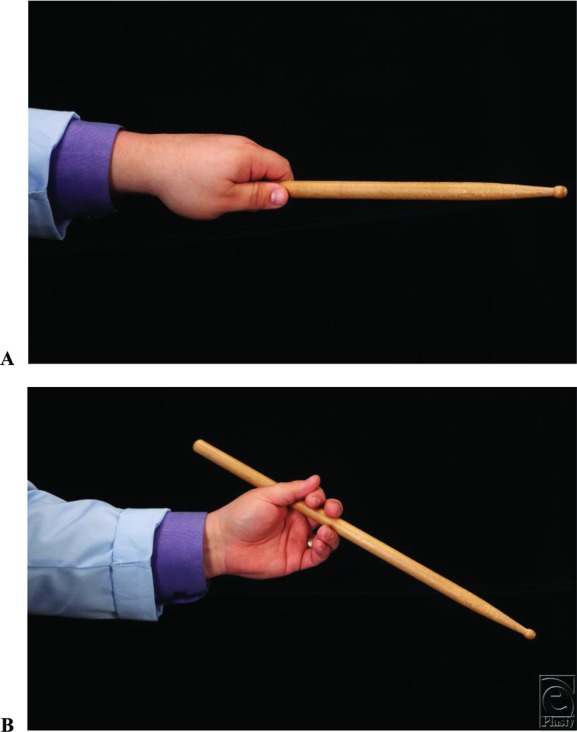
Proper drumstick grip. (a) Demonstration of match grip. The fulcrum is directed between the distal portion of the thumb and the index finger. (b) Demonstration of traditional grip. Note the position of the drumstick through the first web space. Photo credit Maria Ansley.

Percussionists can be quite abusive of their limbs. Aside from superficial damage—bullae from friction or capillary thrombosis from grip—several conditions have been noted to be associated with percussion. The repetitive motion can exacerbate peripheral nerve compressions. Particularly, radial tunnel syndrome can flare from repetitive motion as well as hypertrophy of the extensors. In addition, the median nerve can be compressed from repetitive pronation, as well as forearm finger and wrist flexor hypertrophy. It is not unusual for the lacertus fibrosus to become quite tight from this motion.^2^ Another condition known as drummer's palsy was described in the late 1800s, whereby the extensor pollicis longus tendon can rupture from repetitive friction along Lister's tubercle.^3^ In addition, there are numerous online forums with tips seeking to prevent aches and pains from the abuse of constant pressure and movement.^4^ To understand the case presented, a bit of background on drumming technique is necessary. There are 2 types of grips used in percussion utilizing standard “drumsticks.” The first, called match grip, places the fulcrum at the distal phalanx of the thumb and the proximal interphalangeal joint (PIP) or middle phalanx of the index finger (Fig 4*a*). The traditional grip, for a right-handed player, holds the stick with the left-hand stick butt to the dorsum. It is then directed through the first web space (Fig 4*b*). The fulcrum is then still on the volar PIP to the middle phalanx of the index, but the opposition is at the base of the thumb on the ulnar side. Proper technique requires pulling the hand somewhat into the plane of the fingers, opposite of what is seen with key pinch. This is to say, a CMC arthroplasty could put the digits in a poor position to play.

In this case, the patient presented with a palpable mass at the base of the thumb. The differential diagnosis should be considered on the basis of anatomic layers and cellular type.^5^ In the skin, this could be an epidermal inclusion, sebaceous cyst, or infectious process. Lipoma or angiolipoma can be found in the subcutaneous tissue. Synovial cysts or ganglions could be found arising from the tendon sheath or the joint itself. A bony or cartilaginous tumor would be firmer on palpation. Nerve tumors are a possibility, such as neuromas or schwannomas. As mentioned in the MRI findings, it could have simply been callous formation in the soft tissues. As in this case, vascular tumors are a possibility. These include arteriovenous malformation, vascular tumor, or vascular occlusion with thrombosis.

The appearance of the digital artery in this case resembled a thrombosed artery, like what is seen in HHS. Hypothenar hammer syndrome is a well-described syndrome of the hand characterized by repetitive trauma to the hand resulting in aneurismal change to the ulnar artery, subsequent occlusion, and arterial insufficiency. This is a direct result of the anatomy of the hook of the hamate and its relationship with the ulnar artery. There are also case reports of thenar hammer syndrome, with a similar phenomenon with the radial artery occurring at the trapezium leading to the base of the first metacarpal.^6^ These syndromes manifest as cold intolerance and Raynaud's phenomenon, most affecting the digits on the ulnar or radial sides, depending on the level of reconstitution and patency of the palmar arch from the contralateral artery and arterial dominance. While the treatment is somewhat debated, there is documentation of successful reconstruction with the descending branch of the lateral femoral circumflex artery.^7^ Evidence has shown that arterial reconstruction with arterial grafts appears to maintain patency better in the long term, though the benefit is unclear in a patient with no vascular symptoms, such as is the case with this patient. Furthermore, a study in *Hand* showed that Quick Disabilities of the Arm, Shoulder and Hand (DASH) scores were similar in patients with all methods of reconstruction—ligation, vein graft, and primary repair. In addition, complications and overall improvement rates were similar. The most common complication from treatment was cold intolerance, regardless of the method used.^8^

**Video 1:** Patient one month post mass resection. The patient is playing a live recording for his band's next studio album.

Despite numerous documented episodes of hypothenar hammer, there are no documented episodes of digital thromboses from similar repetitive trauma. This could be multifactorial, though the natural anatomy of the digits likely does not present a compression point with most composite grip activities in contrast with the wrist, which has 2 distinct anatomic areas where this is likely. The digital neurovascular bundles run paramedian, approximately 2 mm volar to the mid-lateral crease and several millimeters from the midline where contact with the bone, underlying tendon or pulleys, or even sesamoid bones is unlikely. In normal anatomy, this is of little consequence. However, in the case of a percussionist who has developed arthritis and undergone CMC arthroplasty with trapeziectomy, the thumb has been pulled out of plane from the digits in order to provide key pinch and function. This causes direct pressure on the digital neurovascular bundle at the base of the proximal phalanx and the metacarpal head. The sum effect could result in the digital artery being at risk for repetitive trauma. This is not to say that other repetitive traumatic activities could not reproduce this phenomenon. In fact, this should increase awareness that digital vessel thrombosis is as susceptible to pseudoaneurysm and thrombosis as the ulnar artery, given repetitive trauma in a workplace or social setting. A careful history and physical is likely to elucidate such a finding and increase the likelihood of appropriate preoperative testing to help rule this in or out.

## SUMMARY

We present a patient with digital artery thrombosis from repetitive trauma. Intraoperative findings suggested arterial thrombosis similar to that found in HHS. We believe this is the first published case of arterial occlusion from this process. Hand surgeons should consider repetitive trauma in occupation or hobbies a source of an atypical mass or vascular phenomenon isolated to the digit.
